# A Healthy Attitude Rural Leaders in TN County Organize to Address Well-Being in Appalachia

**DOI:** 10.13023/jah.0201.08

**Published:** 2020-01-26

**Authors:** Tim Marema, Erin Bouldin

**Affiliations:** Center for Rural Strategies; Appalachian State University

**Keywords:** Appalachia, philanthropy, community, rural health

## Abstract

When it came to formal philanthropy, Grundy County was not on the map. That changed with the 2012 establishment of South Cumberland Community Fund, which serves the plateau portions of Grundy, Franklin, and Marion counties.

It’s lunchtime at Sam’s Corner restaurant in Coalmont, Tennessee. A group of six women is gathered at one of the larger tables, conversing while they await their orders. When the steaming food arrives, the talk slows a bit, but it does not stop. The subject: how to improve community health. And in Grundy County, Tennessee, that means there is a lot to talk about.

“It all goes back to what my mom and dad said,” says Emily Partin, one of women at the large table, referring to efforts to address the county’s difficult health issues. “You have to bloom where you’re planted.”

That’s not always easy. Last year rural Grundy County ranked last in Tennessee in its “health outcomes” in the annual County Health Rankings and Roadmaps report. There are signs of change. In this year’s rankings,^1^ Grundy County moves up two position in its health outcomes, from 95 (last place in the state) to 93.

But at the lunch table, 2 months before the release of the new report, the women aren’t focused on their position on the list. (“For us to move up, someone else has to move down,” Partin says. “And we don’t want to wish that on anybody.”) But they are focused on improving their residents’ health.

They have a lot to show for it. The community has implemented several new programs in recent years because of local leadership, with outside technical assistance and funding at key points along the way. So how does a county that lies near the bottom in Tennessee’s health rankings create a top-tier effort to change?

## HIT THE TRAIL

One indicator of health is residents’ access to recreational facilities. The Mountain Goat Trail Alliance^2^ is converting an old railroad right-of-way into recreational trails. It is not easy to convince people who live in an economically distressed area that trails are the best use of public investment, Partin said. But there’s a direct link to improving community health. “We see that as part of the built environment that’s going to help address some of our health disparities,” she said. “Even though you are a rural county and there are lots of grassy areas, there’s really no place for families to get together and get exercise like pushing a baby stroller.”

Only a third of Grundy County residents say they have access to exercise opportunities, such as walking trails. With easily accessible and family-friendly trails, walking is much more practical. Local people have started competing in a national walking challenge. For example, Tracy City First United Methodist had about 60 people sign up for the challenge last year. Together they logged about 11,000 miles and engaged in friendly competition with other groups.

And the Mountain Goat Trail keeps growing. Grundy County government acquired 17 more miles of railroad right of way in 2018. That will extend the trail all the way to Palmer, a former coal camp that lies just a mile or two from Savage Gulf State Natural Area.

## FAMILIES FIRST

Another set of community efforts has addressed parenting and children. Grundy County was the first rural site (and just the second in the entire state) to create a safe-babies court team.^3^ The program works with parents who have entered the child-welfare judicial process with a child aged 3 years or younger. Rather than taking a merely judicial approach, advocates help families gain access to programs that can improve conditions at home.

“In baby court, even though our focus is on that baby and that family, there’s an even bigger chance that the next baby being born into that family is going to get the benefits of the energy, time, and resources we’ve put into that family,” said Katie Goforth, who manages the AmeriCorps VISTA project at the University of the South and whose background is in behavioral health.

For other parents, Partin has started Discover Together. She calls the program a place-based family co-op for families with children from birth to 5 years old. Twenty families are participating; they meet twice a week for 2–3 hours for multiage activities that mix toddlers, preschoolers, and parents. The multi-age mixing can surprise people. The long-term goal is for parents in the program to share what they learn. “We are hoping that they are going to become the ambassadors that take the information out to others,” Partin said.

## COMMUNITY PHILANTHROPY

When it came to formal philanthropy, Grundy County was not on the map. That changed with the 2012 establishment of South Cumberland Community Fund, which serves the plateau portions of Grundy, Franklin, and Marion counties.

“Previously, there were no local philanthropic resources,” said Jack Murrah, an early proponent of the foundation. A resident of Monteagle, Murrah moved to Grundy County after a career at the Lyndhurst Foundation in Chattanooga.

With professional expertise and community contacts, volunteers found ways to tap community wealth. The University of the South at Sewanee, a liberal arts college located on the plateau a few miles from Grundy County, played a big role. Early support also came from second-home owners and residents who moved to the area for its natural amenities. Since 2012 the South Cumberland Community Fund has awarded more than $500,000 for community-minded projects to 45 different organizations.

The organization provides more than money, Murrah said. “[The fund] supports the nonprofit sector with technical assistance and training,” he said. “They have helped with capacity building and building a network within the nonprofit sector.” The foundation also sponsors training, such as courses through Nashville’s Center for Nonprofit Management.

## TOWN AND GOWN

The University of the South has a long history of involvement with communities on the Cumberland Plateau. Besides helping start and sustain the community foundation, the school also established the Office of Civic Engagement, which connects the university directly to local agencies. The program takes its cues from local institutions, many of which are working on health-related issues, said Jim Peterman, a philosophy professor who also heads the Civic Engagement Office.

“Our orientation for working in the community, right from the outset, was not the traditional service model,” Peterman said. “It has been to work in communities in ways that build capacity for them to achieve their own aspirations.”

The office facilitates student internships, community-focused research, and an AmeriCorps VISTA program with local agencies, among other activities.

## BOTTOM LINE

Despite Grundy County’s improvement in the 2019 rankings, the county remains near the bottom of Tennessee’s 95 counties. So are the programs making a difference?

“What stands out in Grundy County is that they have decided to take action,” said Aliana Havrilla, a community coach with County Health Rankings and Roadmaps. “There’s a core group of people in the community working to improve health outcomes and strengthen the community. And they are reaching out to others to include them in the journey.”
